# Mutations of RagA GTPase in mTORC1 Pathway Are Associated with Autosomal Dominant Cataracts

**DOI:** 10.1371/journal.pgen.1006090

**Published:** 2016-06-13

**Authors:** Jian-Huan Chen, Chukai Huang, Bining Zhang, Shengjie Yin, Jiajian Liang, Ciyan Xu, Yuqiang Huang, Ling-Ping Cen, Tsz-Kin Ng, Ce Zheng, Shaobin Zhang, Haoyu Chen, Chi-Pui Pang, Mingzhi Zhang

**Affiliations:** 1 Joint Shantou International Eye Center, Shantou University & the Chinese University of Hong Kong, Shantou, China; 2 Department of Ophthalmology and Visual Sciences, the Chinese University of Hong Kong, Hong Kong, China; Case Western Reserve University, UNITED STATES

## Abstract

Cataracts are a significant public health problem with no proven methods for prevention. Discovery of novel disease mechanisms to delineate new therapeutic targets is of importance in cataract prevention and therapy. Herein, we report that mutations in the RagA GTPase (*RRAGA*), a key regulator of the mechanistic rapamycin complex 1 (mTORC1), are associated with autosomal dominant cataracts. We performed whole exome sequencing in a family with autosomal dominant juvenile-onset cataracts, and identified a novel p.Leu60Arg mutation in *RRAGA* that co-segregated with the disease, after filtering against the dbSNP database, and at least 123,000 control chromosomes from public and in-house exome databases. In a follow-up direct screening of *RRAGA* in another 22 families and 142 unrelated patients with congenital or juvenile-onset cataracts, *RRAGA* was found to be mutated in two unrelated patients (p.Leu60Arg and c.-16G>A respectively). Functional studies in human lens epithelial cells revealed that the *RRAGA* mutations exerted deleterious effects on mTORC1 signaling, including increased relocation of RRAGA to the lysosomes, up-regulated mTORC1 phosphorylation, down-regulated autophagy, altered cell growth or compromised promoter activity. These data indicate that the *RRAGA* mutations, associated with autosomal dominant cataracts, play a role in the disease by acting through disruption of mTORC1 signaling.

## Author Summary

A group of guanine nucleotide-binding molecules called Rag GTPases are known to play a crucial role in regulation of mTORC1 signaling cascade. In the current study, whole exome sequencing has led to the identification of the RagA GTPase (*RRAGA*) gene for cataracts and we proceeded to study properties of the RRAGA protein. We captured and sequenced the whole exome for four affected patients from a family with autosomal dominant juvenile-onset posterior cataracts, and found a novel rare mutation in RagA GTPase (*RRAGA*). To validate this finding, we then sequenced more families and patients, and observed *RRAGA* mutations in unrelated patients with related phenotypes, suggesting that *RRAGA* could be mutated in congenital and juvenile-onset cataracts. We further demonstrated supporting evidence that in human lens epithelial cells the *RRAGA* mutations exerted deleterious effects on relocation of RRAGA to the lysosomes, mTORC1 phosphorylation, autophagy and cell growth. This study gives important new insight into the roles of *RRAGA* and mTROC1 signaling in the etiology of cataracts.

## Introduction

Cataracts refer to the clouding of the crystalline lens, and is a significant public health problem [1]. Preventative measures for cataracts are critical due to insufficient availability of cataract surgery in much of the world [2] According to the age at onset, cataracts can be classified as congenital, juvenile, presenile, or senile (age-related) [3]. Autosomal dominant cataracts are the most common form of hereditary cataracts with high clinical and genetic heterogeneity [4]. Fewer than twenty causative genes for human autosomal dominant cataracts have been identified, almost half of which are crystallin genes [4]. However, more than half of the familial dominant cataract cases have unknown genetic causes [5], the molecular mechanism of cataracts has not been fully elucidated, and there is no proven method of prevention for cataracts. Deciphering the genetics of hereditary cataracts will not only improve our knowledge of the disease, but also help identify new strategies for prevention and therapy of cataracts. For example, a recent breakthrough in gene mapping, revealing a mutated lanosterol synthase to be a causative gene for autosomal recessive congenital cataracts, has led to the use of lanosterol to reverse protein aggregation in adult onset cataracts in dogs [6].

In addition to traditional approaches of linkage and candidate gene studies that continue to contribute to genetic studies of cataracts [7], next-generation sequencing technologies have advanced our understanding of genes and mutations that cause hereditary cataracts [8]. A target sequencing of 115 genes in sixty-five syndromic and non-syndromic congenital cataract patients showed that the technology can provide better diagnostic and management outcomes [7]. Our previous study also demonstrated that whole exome sequencing (WES) could offer a rapid and cost-effective diagnosis for autosomal dominant congenital cataracts [9].

We performed WES on members of a family with autosomal dominant progressive posterior subcapsular cataracts, and with no known autosomal dominant cataract gene mutations, and identified a novel missense p.Leu60Arg mutation in the Ras-related GTP-binding protein A (also called RagA GTPase, *RRAGA*) gene [MIM 612194], involved in the mTORC1 pathway. The p.Leu60Arg mutation and a 5’-UTR mutation were observed in two unrelated patients from a direct screening of 22 families and 142 unrelated patients with congenital or juvenile onset cataracts. Functional analysis showed deleterious effects of the *RRAGA* mutations on the mTORC1 pathway in human lens epithelial cells. Our findings thus indicate that the *RRAGA* mutations are associated with autosomal dominant cataracts through disruption of the mTORC1 pathway, a substantial modulator of ageing and age-related human diseases [10].

## Results

### Identification of the *RRAGA* p.Leu60Arg mutation in a family with juvenile-onset progressive posterior subcapsular cataracts

**Fig 1:**
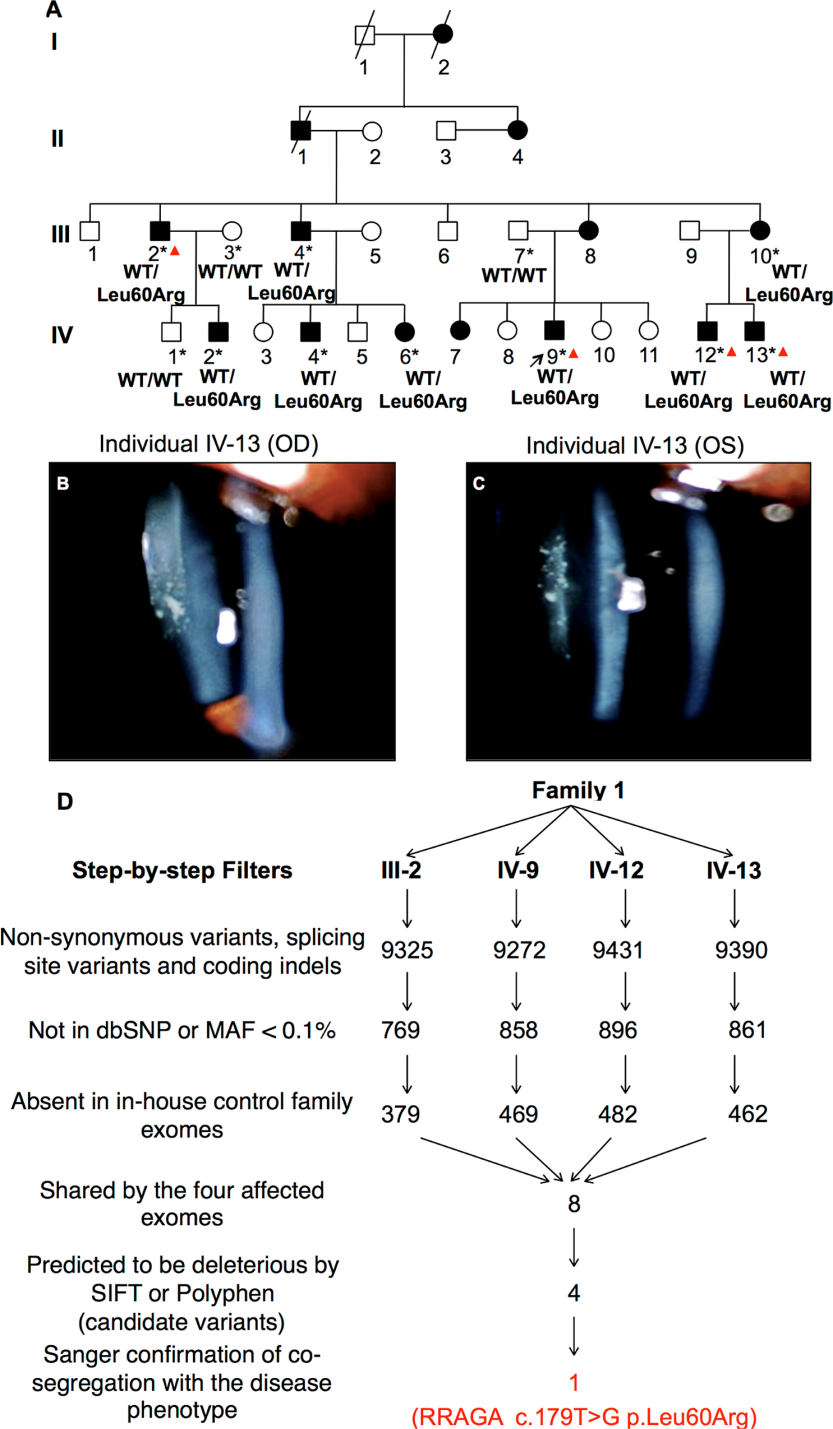
Identification of a *RRAGA* missense mutation in a four-generation Chinese family with autosomal dominant juvenile-onset progressive posterior subcapsular cataracts. (**A**) Pedigree of Family 1. Squares represent males, circles females, black symbols affected individuals, and white symbols unaffected individuals. All affected family members have juvenile-onset progressive posterior subcapsular cataracts. The arrow denotes the proband. Asterisks denote individuals with DNA collected from blood samples. Red triangles denote individuals from whom DNA was subjected to WES in the current study. Genotypes are indicated beneath the individual symbols. The heterozygous missense mutation p.Leu60Arg shows complete co-segregation with the progressive posterior subcapsular cataract phenotype. The results of slit-lamp examination for IV-13 in both OD (**B**) and OS (**C**) demonstrate deposition of dot-like opacity underneath the posterior capsule of the lens in affected family members. The age at onset in most of these affected individuals is between adolescent and early adulthood, suggesting a juvenile disease onset. (**D**) Step-by-step filtering strategy in Family 1 for the four affected individuals subjected to WES. The filtering criteria are given on the left side of the workflow. The number of variants that remain after each filtering step is shown in the workflow. The Four candidate variants were obtained as shown in Table 2. Among these variants a heterozygous missense mutation c.179T>G (p.Leu60Arg) in *RRAGA* was identified after Sanger confirmation of co-segregation.

To identify mutated genes for autosomal dominant cataracts, we investigated a four-generation Chinese Han family with bilateral juvenile-onset progressive posterior subcapsular opacity (Family 1, **Fig 1 and Table 1**). WES was performed in four affected (III-2, IV-9, IV-12 and IV-13) family members of Family 1 (**Fig 1A**). The four affected individuals were expected to share about 0.7% (1/128) of all variants by descent [11]. The variants called from these exomes are summarized in S1 and S2 Tables. 23 known autosomal dominant cataract genes had a 5X minimal exon depth (**S3 Table**), and thus were 100% covered in the four exomes, which ensured low false negative rate in detection of causing variants in these genes. We further screened all candidate genes from the cat-map database (http://cat-map.wustl.edu). No rare variant in these genes was found to detect in all of the four affected exomes. We have thus excluded known disease-causing genes as the underlying cause of cataracts in this family.

**Table 1:** Clinical features of congenital or juvenile-onset cataract patients with *RRAGA* mutations in the current study.

Patient (Mutation)	Sex	Age at recruitment	Age at diagnosis	Visual acuity before surgery		Visual acuity after surgery		Details of cataract phenotype	Other ocular symptoms
				OD	OS	OD	OS		
Family 1									
III-2 (Leu60Arg)	Male	57	20	NA	NA	NA	NA	Bilateral opacity	Secondary glaucoma
III-4 (Leu60Arg)	Male	50	20	NA	NA	NA	NA	Bilateral opacity	None
III-10 (Leu60Arg)	Female	40	15	NA	NA	NA	NA	Bilateral opacity	None
IV-2 (Leu60Arg)	Male	21	13	NA	NA	0.6	0.6	Bilateral opacity	None
IV-12 (Leu60Arg)	Male	18	14	0.3	0.02	0.5	0.3	Bilateral posterior subcapsular opacity	None
IV-9 (Leu60Arg)	Male	17	13	0.2	0.15	0.5	0.4	Bilateral posterior subcapsular opacity	None
IV-4 (Leu60Arg)	Male	17	15*	0.6	0.6	NA	NA	Bilateral posterior subcapsular opacity	None
IV-13 (Leu60Arg)	Male	13	13*	1.0	0.06	NA	NA	Bilateral posterior subcapsular opacity	Amblyopia
IV-6 (Leu60Arg)	Female	13	13*	1.0	1.0	NA	NA	Bilateral posterior subcapsular opacity	None
Unrelated patients									
CC38 (Leu60Arg)	Female	24	22	0.25	0.25	0.8	0.5	Bilateral anterior and posterior subcapsular opacity	None
CC19 (c.-16G>A)	Male	15	13	0.3	0.15	0.4	0.2	Bilateral nuclear opacity	None

NA: not available

*IV-4, IV-13 and IV-6 had not yet received cataract surgery. The lens opacity in IV-13 and IV-6 remained asymptomatic at the time of clinical examination.

The variants called from the exome data were then analyzed using a stepwise filtering method (**Fig 1D**), and a total of four final candidate variants were obtained (**Table 2**). Sanger sequencing of these candidate variants in Family 1 eventually identified a novel heterozygous missense mutation c.179T>G (p.Leu60Arg) in the Ras-related GTP-binding protein A (*RRAGA*) gene (Refseq accession numbers: mRNA NM_006570.4 and protein NP_006561.1) (**S1 Fig**). The mutation was observed in all recruited affected individuals, but absent in recruited unaffected individuals, confirming co-segregation with the posterior cataract phenotype in Family 1 (**Fig 1A** and **S1 Fig**). Parametric linkage analysis obtained a 2.975 LOD score between the variant with the posterior cataract phenotype.

**Table 2:** Characteristics of the four candidate variants resulting from filtering of exome sequencing data for Family 1.

No.	Chromosomal position^a^	Gene symbol	mRNA	cDNA change	Protein change	Functional prediction		dbSNP	MAF^d^	Co-segregation in Family 1
						SIFT^b^ (score)	Polyphen^c^ (score)			
1	chr9:19049836	*RRAGA*	NM_006570	c.179T>G	p.L60R	Deleterious (0.00)	Probably damaging (0.996)	NA	NA	Confirmed
2	chr9:86518180	*KIF27*	NM_017576	c.1253C>G	p.A418G	Deleterious (0.01)	Probably damaging (0.999)	rs192546948	0.08%	Excluded^e^
3	chr16:30409074	*ZNF48*	NM_001214906	c.503A>G	p.Y168C	Deleterious (0.03)	Benign (0.016)	NA	NA	Excluded^e^
4	chr19:57334187	*PEG3*	NM_001146184	c.499C>T	p.R167W	Deleterious (0.03)	Possibly damaging (0.835)	rs547466388	0.08%	Excluded^e^
		*ZIM2*	NM_001146326	c.124C>T	p.R42W	Deleterious (0.00)	Probably damaging (0.995)	rs547466388	0.08%	Excluded^e^

NA, not available.

^a^Chromosomal positions are given according to the UCSC hg19 reference assembly.

^b^SIFT scores ≤ 0.05 were interpreted as damaging and scores > 0.05 were tolerated.

^c^PolyPhen scores between 0.85 and 1.00 were interpreted as probably damaging, scores between 0.2 and 0.85 were possibly damaging, and scores between 0 and 0.2 were benign.

^d^MAFs are according to the 1000 Genomes Project data.

^e^Sanger sequencing validation results can be found in S5 Table.

### *RRAGA* mutations in additional patients with congenital or juvenile-onset cataracts

**Fig 2:**
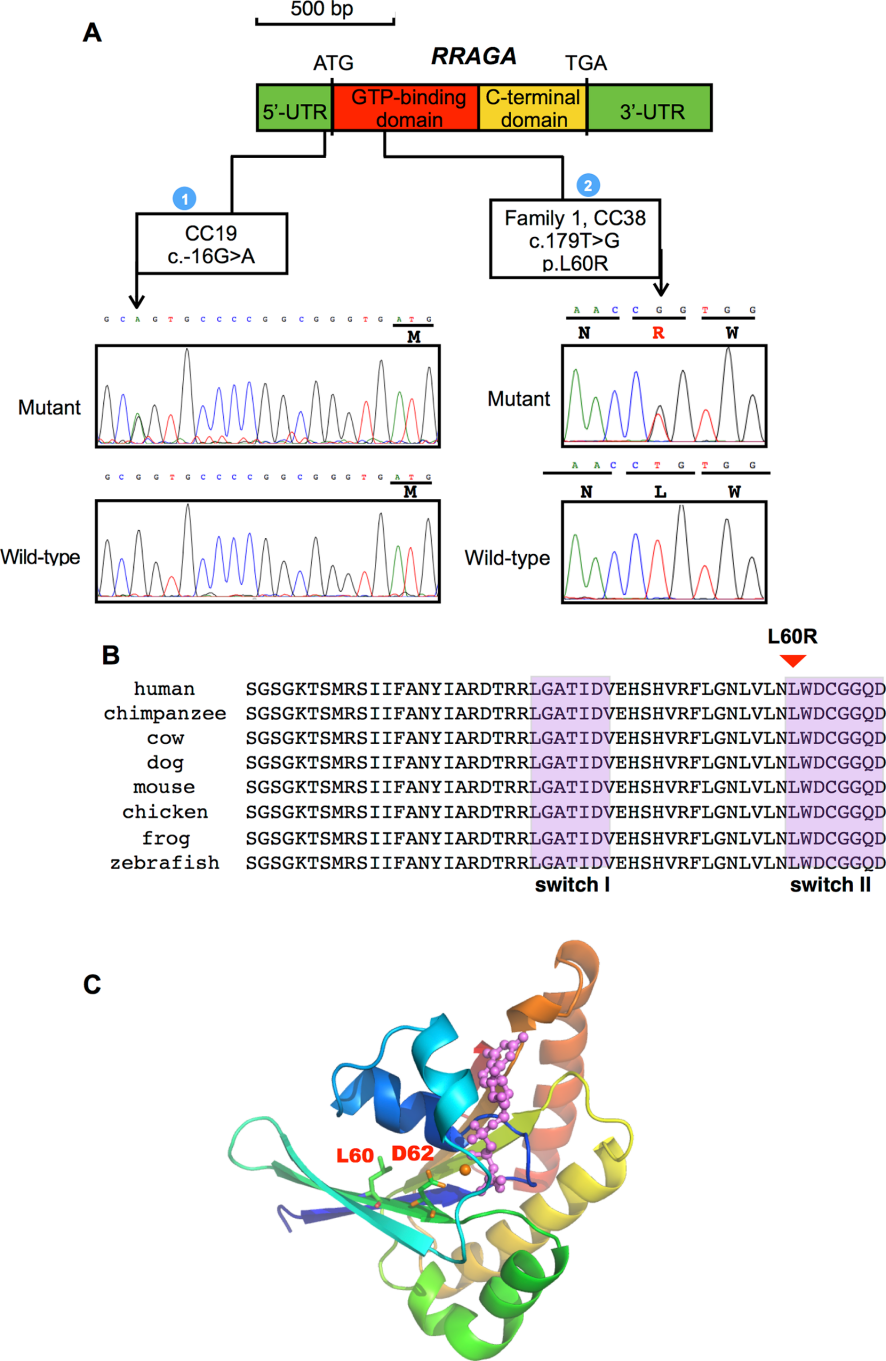
*RRAGA* mutations identified from congenital or juvenile-onset cataract patients. (**A**) The genomic features of the *RRAGA* gene and the domains in the encoded protein. *RRAGA* contains a single exon encoding a GTP-binding domain (red) and a C-terminal domain (yellow). The UTR regions are shown in green. Frequencies of mutations and patients with mutations are indicated as numbers in blue filled circles. The p.Leu60Arg mutation was observed in affected of Family 1 and unrelated patient CC38, while c.-16G>A was found in unrelated patient CC19. (**B**) Protein sequence alignment of RRAGA orthologs in vertebrates. The switch I and II regions in the GTP-binding domain are shaded purple [12]. The conserved leucine residue at codon 60 mutated in affected patients, indicated by a red triangle, is located within switch II. (**C**) Protein homology modeling of the GTP-binding domain of RRAGA. The GTP molecule is shown in purple, and the Mg^2+^ ion in orange. The L60 residue is within the switch II region responsible for mTORC1 activation [12].

The human RRAGA gene has a single exon. We next screened *RRAGA* for possible mutations in probands of 22 families with autosomal dominant cataracts, and 142 unrelated patients with congenital or juvenile-onset cataracts (**Fig 2A**). The coding region, 5’-UTR and 100 bp into the 3’-UTR of *RRAGA* was screened in these individuals using Sanger sequencing. Notably, *RRAGA* p.Leu60Arg was observed in an unrelated patient with juvenile-onset anterior and posterior subcapsular cataracts (CC38) (**S2 Fig and Table 1**). We further reconstructed the haplotype that contains the alternative allele G of RRAGA c.179T>G by selecting 10 coding SNVs that are heterozygous in at least one of the four affected exomes (**S6 Table**) and containing the alternative allele G of RRAGA c.179T>G. These SNVs were then genotyped in the four affected family members and the simplex CC38 patient by using Sanger sequencing. At least two differences were found between haplotypes containing the alternative allele G of RRAGA c.179T>G between Family 1 and SCC38. In addition, a novel heterozygous c.-16G>A mutation in the *RRAGA* 5’-UTR was identified in an unrelated patient (CC19) with congenital nuclear cataracts (**Fig 2A, S2 Fig and Table 1**). The c.-16G>A mutation were absent in at least 12,3000 control chromosomes from the 1000 Genomes Project, ESP, the exome array design data, the ExAC database, 125 in-house control exomes, and Sanger sequencing results of 1,018 control individuals.

### Homology modeling of the RRAGA GTP-binding domain

We then analyzed the possible functional impact of the p.Leu60Arg mutation, on the RRAGA protein, by using homology modeling (**Fig 2B and 2C**). The conserved leucine residue at codon 60 was located in switch II, a region responsible for activation of RRAGA and subsequent activation of mTORC1, and is spatially close to the magnesium (Mg^2+^)-binding site at codon 62 [12]. Therefore, the replacement of leucine with arginine in the mutation was likely to affect the activation of RRAGA and its downstream pathway.

### Expression of RRAGA in human lens epithelia

**Fig 3:**
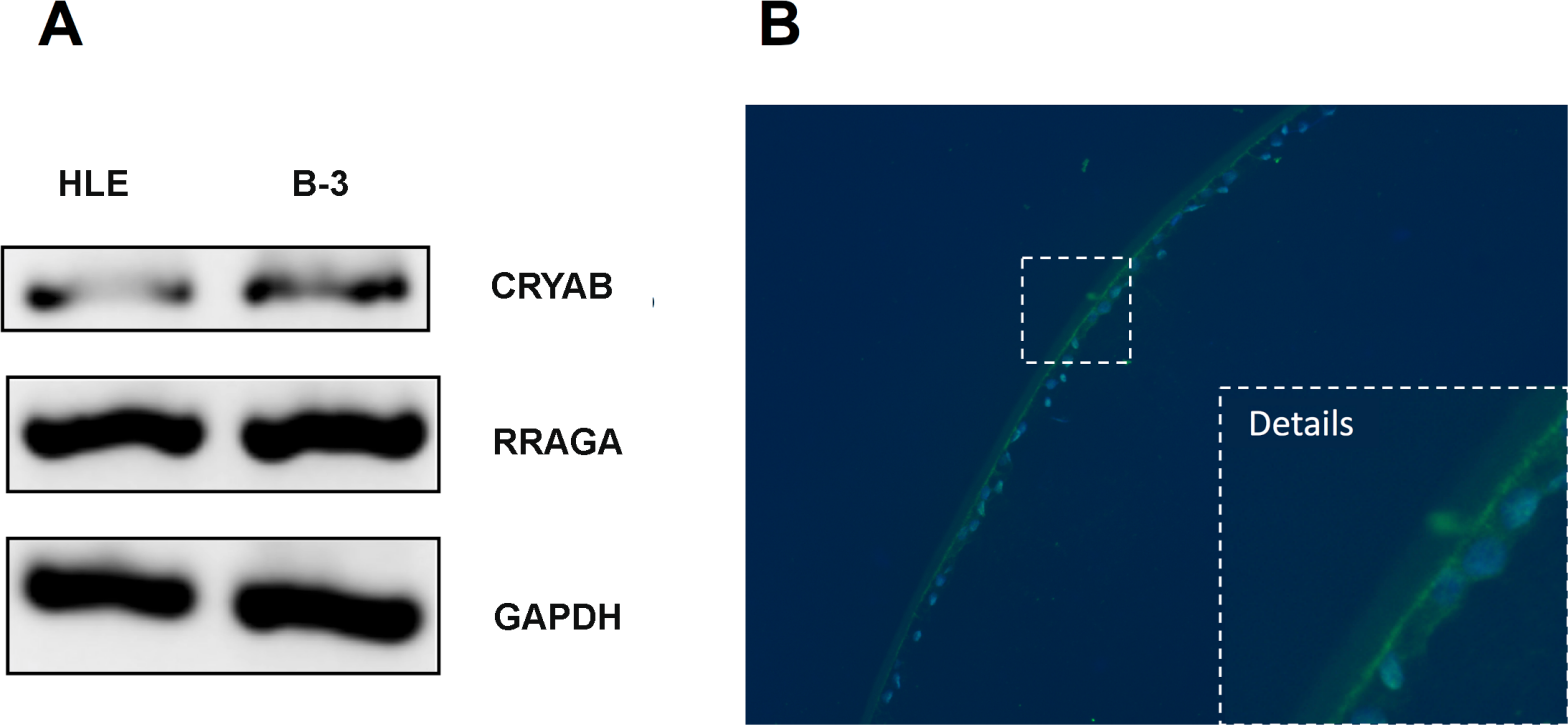
RRAGA is expressed in human lens epithelial cells. (**A**) RT-PCR of *RRAGA* in primary HLE and B-3 cells. *CRYAB* as a lens epithelial marker and *GAPDH* as a housekeeping gene are also shown. (**B**) Immunofluorescence of RRAGA (green) in the human lens. Nucleus is stained with DAPI (blue).

Human lens epithelia (HLE) regulate most of the homeostatic functions of the lens, serve as the progenitors of lens fibers, and are important in maintaining the transparency of the lens [13]. We thus further examined expression of *RRAGA* in primary HLE and B-3 cells. Reverse-transcription polymerase chain reaction (RT-PCR) showed that *RRAGA* was expressed in primary HLE and B-3 cells (**Fig 3A**). Expression of αB-crystallin (*CRYAB*), was highly expressed in human HLE was also shown as a tissue marker (**S4 Fig**). Expression of RRAGA in B-3 cells was further confirmed using Western blot (**S4 Fig**). Immunofluorescence staining for RRAGA in archived human eyeballs also confirmed expression of RRAGA in the lens (**Fig 3B**). The expression level of RRAGA is the highest in the lens, lower in the retina, and the lowest in the cornea (**S5 Fig**).

### Deleterious effects of *RRAGA* p.Leu60Arg on mTORC1 pathways in B-3 cells

**Fig 4:**
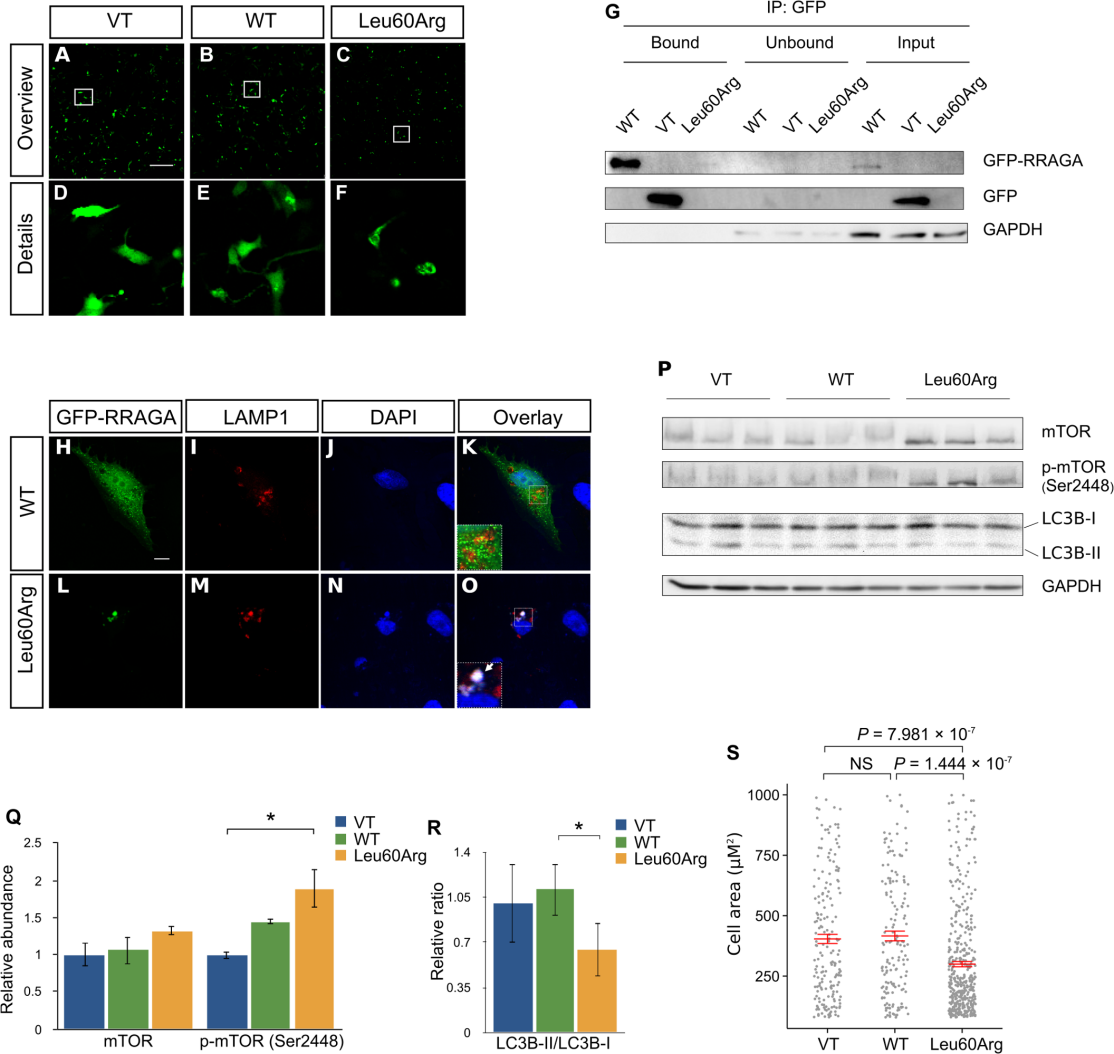
Effect of the p.Leu60Arg mutation in human lens epithelial cells. (**A-F**) Live imaging of B-3 cells transfected with the GFP vector (VT), GFP-RRAGA wild-type (WT) or p.Leu60Arg mutant (Leu60Arg). Arrow denotes reduced cell area of in cells expressing Leu60Arg. Scale bar indicates 250 μm. (**G**) GFP-trap immunoprecipitates (IP) from transfected B-3 cells analyzed by immunoblotting using antibody against GFP. Results of total cytosol (input), unbound protein and immunoprecipitated (bound) protein are shown. GAPDH is used as a loading control. (**H-O**) Co-transfection of RFP-LAMP1 with GFP-RRAGA in B-3 cells. Arrows denote co-localization between RRAGA Leu60Arg and LAMP1. RRAGA Leu60Arg was mostly located within the lysosomes. Scale bar indicates 10 μm. (**P**) Immunoblotting of mTOR, p-mTOR (Ser2448), and the autophagy marker LC3B in B-3 cells expressing GFP, or RRAGA WT or Leu60Arg. (**Q, R**) Quantification of the immunoblotting results of mTOR and LC3B-II/LC3B-I ratio. The phosphorylated form, p-mTOR (Ser2448) was upregulated in cells transfected with RRAGA Leu60Arg than those with RRAGA WT. In line with such mTOR changes, a remarkable reduction of LC3B-II/LC3B-I ratio was found in cells expressing RRAGA Leu60Arg compared to those expressing RRAGA WT. Data are presented as the mean plus S.E.M. *P* values were determined by Student's t test. (**S**) Measurement of the area of B-3 cells expressing GFP (analyzed cell n = 183), or GFP-RRAGA WT (analyzed cell n = 158) or RRAGA Leu60Arg (analyzed cell n = 463). Mean plus S.E.M. is shown. *P* values were determined by the Mann-Whitney U test. *, *P* < 0.05.

We evaluated the effects of the p.Leu60Arg mutation on RRAGA by overexpressing GFP-RRAGA in B-3 human lens epithelial cells (**Fig 4**). Our data indicated that expression of GAPDH is relatively stable in these cells with different treatment (by fixing the total amount of loading samples), and is eligible to be used as an internal control as reported previously [14,15]. Wild-type RRAGA was mostly dispersed in the cytoplasm and nucleus (**Fig 4B, 4E, and 4K**). In contrast, the RRAGA p.Leu60Arg mutant (Leu60Arg) was predominantly localized in the cytoplasm of the transfected cells (> 90%) (**Fig 4C, 4F and 4O)**. Moreover, when analyzed using immunoblotting, RRAGA Leu60Arg was dramatically reduced in the cytosol compared to wild-type RRAGA, even after enrichment by immunoprecipitation with GFP antibody (**Fig 4G**). The reduced level of cytosolic RRAGA Leu60Arg could be associated with abnormal lysosome function, since the activated form of RRAGA is normally recruited to the lysosomes [12]. We thus further looked into the co-localization of RRAGA with the lysosome marker lysosome-associated membrane protein 1 (LAMP1) by co-expressing the two in B-3 cells. Wild-type RRAGA was mostly dispersed in the cytoplasm and nucleus (**Fig 4H–4K**), with only a small fraction co-localized with LAMP1. In contrast, the RRAGA Leu60Arg mutant was mostly co-localized with lysosomes near the nucleus (**Fig 4L–4O**), indicating that an increased level of activated RRAGA. Immunoblotting was further used to evaluate the impact of RRAGA Leu60Arg mutant on the downstream mTORC1 pathway. Our results showed that mTOR and its phosphorylated form p-mTOR (pSer2448) were up-regulated in cells expressing RRAGA Leu60Arg compared to those expressing the wild-type RRAGA (**Fig 4P and 4Q**). In addition, the autophagy marker LC3B was also affected by RRAGA Leu60Arg, which caused a lower LC3B-II to LC3B-I ratio in cells expressing Leu60Arg compared to those expressing wild-type RRAGA (*P* < 0.05) (**Fig 4P and 4R**). This suggested that autophagy was down-regulated by overexpression of the RRAGA Leu60Arg mutant. Noteworthy, on average a significantly smaller cell area was observed in B-3 cells expressing RRAGA Leu60Arg (**Fig 4S**) compared to those expressing GFP (*P* = 7.981 × 10^−7^) or wild-type RRAGA (*P* = 1.444 × 10^−7^), while no significant difference in cell area was found between cells expressing GFP and wild-type RRAGA (*P* > 0.05), indicating RRAGA Leu60Arg could be associated with altered cell growth.

### *RRAGA* c.-16G>A is associated with reduced promoter activity

**Fig 5:**
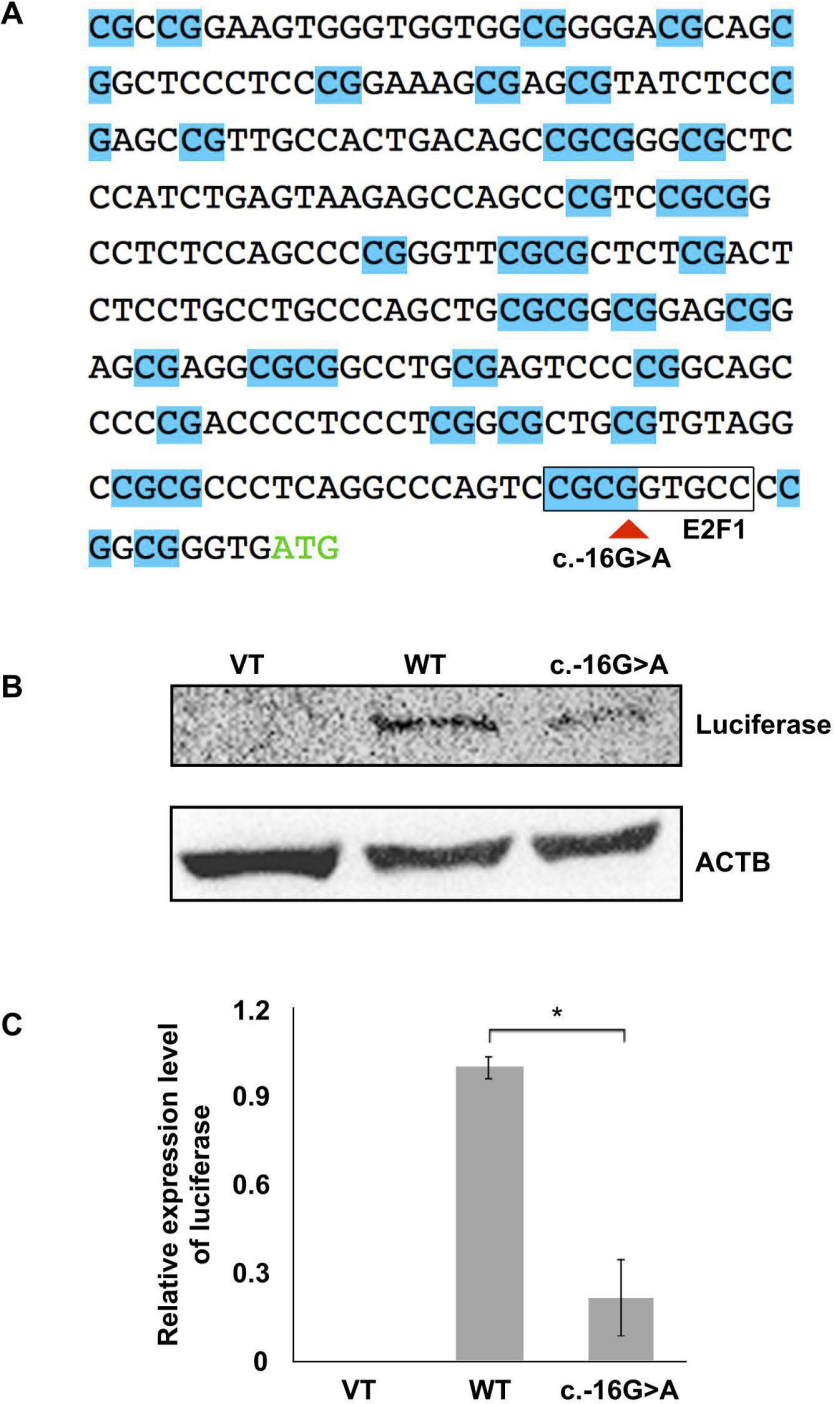
The c.-16G>A mutation in the 5’-UTR of *RRAGA* is associated with reduced promoter activity. (**A**) The 5’-UTR and start codon of *RRAGA*. The start codon (ATG) is shown in green. The *RRAGA* 5’-UTR is predicted to be associated with promoter activity (Ensembl feature: ENSR00001469646) in the Ensembl database, and is enriched with CpG dinucleotides (highlighted in blue). The c.-16G>A mutation indicated by a red triangle, overlaps with a CpG dinucleotide and a predicted binding site for transcription factor E2F1, which regulates mTORC1 signaling [17]. The mutant A allele was predicted by PROMO [16] to abolish the binding site. (**B**) The c.-16G>A mutant is associated with reduced luciferase reporter expression compared with the wild-type (WT) in B-3 cells. B-3 cells were transfected with pGL3-enhancer vectors with no RRAGA 5’-UTR (VT), or with the wild-type RRAGA 5’-UTR (WT), or 5’-UTR c.-16G>A mutant. The expression level of luciferase reporter was determined using immunoblotting. (**C**) Quantification of the immunoblotting results of luciferase expression. Data are presented as the mean plus S.E.M. *P* values were determined by Student's t test. *, *P* < 0.05.

The *RRAGA* 5’-UTR is predicted to have promoter activity in the Ensembl database (**Fig 2**). In addition, the c.-16G>A mutation was predicted to abolish a CpG dinucleotide and a binding site for E2F1 by using PROMO [16], a transcription factor that regulates mTORC1 signaling [17] (**Fig 5A**). To determine the impact of the mutation on promoter activity, the wild-type RRAGA 5’-UTR and 5’-UTR with the c.-16G>A mutant were individually cloned into the pGL3-enhancer luciferase vector, and used to transfect B-3 cells (**Fig 5B**). We used anti-luciferase antibody and immunoblotting to directly assess promoter activity instead of indirect methods such as luciferase substrate. Our results showed that expression of the luciferase reporter, containing the 5’-UTR with the mutant A allele, was significantly lower than that containing the wild-type G allele (~80% reduction, *p* < 0.05, **Fig 5C**). Therefore, the c.-16G>A mutation probably affected the promoter activity.

## Discussion

In the current study, using WES, we identified a novel heterozygous missense p.Leu60Arg mutation in *RRAGA* that co-segregated with progressive juvenile-onset posterior subcapsular cataracts. *RRAGA* was mutated in patients with congenital or juvenile-onset cataracts. Functional studies showed deleterious effects of these *RRAGA* mutations on mTORC1 pathway in human lens epithelial cells. Our results thus show that *RRAGA*, which encodes the RagA GTPase, is a novel gene associated with autosomal dominant cataracts, and implicate a novel mechanism involving the mTORC1 pathway that is different from our prior knowledge of cataracts.

The molecular genetics and clinical presentations of autosomal dominant cataracts are highly heterogeneous. Posterior cataracts are one of the most severe subtypes that affect visual outcome [18]. Mutations that cause autosomal dominant progressive posterior cataracts have been reported in *CHMP4B* [19] and *PITX3* [20], but the clinical phenotypes are different as those in Family 1. In this study, we identified a *RRAGA* Leu60Arg mutation in all affected individuals of the family, all of whom had posterior subcapsular cataracts, characterized by a progressive accumulation of dot-like opacity, since adolescence or early adulthood (**Table 1**). The youngest affected member of the pedigree (IV-13, 13 years old at first diagnosis) had small dot-like opacity beneath the posterior capsule, with her vision acuity unaffected (1.0), suggesting that asymptomatic opacity could possibly exist in these affected patients before diagnosis. Noteworthy, a highly consistent phenotype-genotype correlation was found through observation of the *RRAGA* p.Leu60Arg in an unrelated patient with juvenile-onset anterior and posterior subcapsular cataracts (**S3 Fig**). Another deleterious mutation was found in the RRAGA 5’-UTR from an unrelated patient with congenital nuclear cataracts, suggesting that clinical heterogeneity also applies. The *RRAGA* mutations were not detected in 2,036 control chromosomes from 1,018 controls using Sanger sequencing, and 125 in-house control exomes, all of whom are from the local population. In addition, the mutation is absent in the ExAC data which contain 4,327 East Asians. Therefore, *RRAGA* mutations are very rare in the general populations. But mutation c.179T>G was found to be heterozygous both in a family and an unrelated simplex patient with juvenile onset posterior cataracts, and another *RRAGA* mutation, c.-16G>A, was found in a patient with bilateral congenital nuclear cataracts. In addition, haplotype analysis showed that haplotypes containing allele G of *RRAGA* c.179T>G were different in Family 1 and the unrelated simplex SCC38, suggesting that the mutation was unlikely inherited from a common founder. Taken together, these findings show involvement of *RRAGA* as a mechanism for autosomal dominant cataracts.

*RRAGA* is a small gene with a single exon spanning 1.65 KB on chromosome 9q14.1, and encodes a guanine nucleotide-binding Rag GTPase that regulates the mechanistic target of rapamycin complex 1 (mTORC1). This complex is a central controller of cell metabolism, growth, proliferation, and survival [21–23]. Activated mTORC1 promotes anabolic processes, including protein and lipid synthesis, and inhibits catabolic ones, such as autophagy [24]. In the current study, the conserved Leu60 residue mutated in juvenile-onset cataract patients is in the switch II region responsible for RRAGA activation [12], and is spatially close to the Mg^2+^ binding site. Therefore, one potential impact of this mutation is a change in the affinity to Mg^2+^, which is required for RRAGA binding to GTP and subsequent activation. Consistent with such a prediction, RRAGA Leu60Arg was mainly located in the lysosomes and was remarkably reduced in the cytosol. One possible explanation is that RRAGA Leu60Arg triggers a higher level of activated Rag GTPases, which are then relocated to the lysosomes. Our data showed an increased mTOR phosphorylation level at Ser2448 in cells expressing RRAGA Leu60Arg. Copp *et al*. have demonstrated that mTORC1 contains primarily mTOR phosphorylated on Ser2448 [25]. Thus, in our study, increased p-mTOR Ser2448 indicates that RRAGA Leu60Arg up-regulates the mTORC1 phosphorylation level. The upregulated mTORC1 phosphorylation indicates that the colocalization of mutant RRAGA with lysosomes is more likely due to RRAGA activation instead of degradation, although it is still possible that long term accumulation of RRAGA in the lysosmes may also lead to protein degradation. It is noted that the downstream LC3B-II/LC3B-I ratio is reduced, suggesting autophagy could be down-regulated. Therefore, RRAGA is more likely to be activated than degraded.

The discovery of association between mTORC1 pathway gene *RRAGA* and autosomal dominant cataracts has important implications for our understanding of cataract disease and ageing. First, deficiencies in regulation of mTORC1 have been found in human diseases and animal models. A germ line mutation in one of the Ragulator proteins causes a primary immunodeficiency human syndrome [26]. Deletion of *Rraga* in mouse embryos causes embryonic death, loss of mTORC1 activity, and severe growth defects, and inducible knockout of *Rraga* in adult mice is also lethal [27]. A constitutively-activated form of RRAGA causes de-regulation of mTORC1, and impairs autophagy in neonatal mice [28]. Results of these studies indicate that over-activation and suppression of RRAGA, and in turn mTORC1, could contribute to diseases. However, to our knowledge, prior to the current study, no *RRAGA* mutation has been clearly associated with any human disease. In our study, RRAGA p.Leu60Arg that up-regulated mTORC1 is associated with juvenile onset posterior cataracts, while c.-16G>A that reduces promoter activity is associated with congenital nuclear cataracts. Our findings indicate the importance of mTORC1 pathway in maintenance of lens transparence, and its disruption might lead to cataract development. Second, the serine/threonine-protein kinase mTOR is a key modulator of ageing and a variety of age-related diseases [10]. Given that senile cataracts are a common age-related disease, the progressive opacification of the crystalline lens in patients with *RRAGA* mutations might be considered as a phenotype of premature ageing that occurs specifically in the crystalline lens. In addition, our findings also revisit the possibility of nutritional modulation of cataracts [2]. Rag GTPases have been known to play an important role in coupling the cellular nutritional signal to the mTORC1 pathway [28]. Third, the role of mTORC1 as a therapeutic target has been intensively investigated preclinically for human diseases, such as cancer [29] and cardiovascular diseases [30]. Hence our findings could be potentially helpful for developing new strategies for cataract prevention and therapy.

We also note that the up-regulation of mTORC1, triggered by RRAGA Leu60Arg, leads to down-regulation of autophagy. Autophagy is involved in a variety of diseases, such as cancer, neurodegenerative diseases, cardiomyopathy and type II diabetes [31,32]. Autophagy has recently been found to be a potentially important process in lens development and cataract formation [33]. Tissue-specific knockout of autophagy-related genes *Atg5* and *Pik3c3* in the lens causes cataracts [34]. Mutations in the autophagy-regulating gene encoding FYVE and coiled-coil domain containing 1 (*FYCO1*) cause autosomal recessive congenital cataracts [35]. Therefore, *RRAGA* mutations probably cause autosomal dominant cataracts by disrupting autophagy. These findings are in line with the substantial role of autophagy in lens development and cataract formation.

In summary, our study identifies mutations in the Rag GTPase *RRAGA*, in the mTORC1 pathway, to be associated with autosomal dominant cataracts, and provides supporting functional evidence. These findings should help advance our understanding of cataract etiology by implicating the role of *RRAGA* and the mTORC1 pathway as associated factors and potential therapeutic targets of cataracts.

## Materials and Methods

### Ethics statement

This study was approved by the Ethics Committee of the Joint Shantou International Eye Center, and was conducted in accordance with the Declaration of Helsinki. Written consent was obtained from each participating subject after explanation of the nature of the study.

### Sample collection and clinical examination

A total of twelve subjects from a Han Chinese family (Family 1, **Fig 1A**) were recruited at the Joint Shantou International Eye Center, Shantou, China. All affected members of Family 1 had bilateral juvenile-onset progressive posterior subcapsular cataracts (**Fig 1B and 1C, Table 1 and S5 Table**). The age of disease onset in most of the affected family members was between adolescence and early adulthood. None of the family members had any other genetic eye disease or syndrome at diagnosis. Twenty-two families with autosomal dominant cataracts, and 142 unrelated patients with congenital or juvenile-onset cataracts, as well as 1,018 unrelated controls with no sign or family history of congenital or juvenile-onset cataracts were recruited from using the same criteria as described above after comprehensive ophthalmic examination. Peripheral blood was collected from all participants, and genomic DNA was extracted by using the QIAmp Blood kit (Qiagen, Hilden, Germany).

### Whole exome capture and sequencing

Genomic DNA (3 μg per individual) from four affected individuals (III-2, IV-9, IV-12 and IV-13) was taken for WES by Macrogen Technologies (Seoul, Korea). The whole exome was captured by an Agilent SureSelect All Human Exon v4.0 kit (Santa Clara, US.), and sequenced on an Illumina HiSeq 2000 (Illumina, Hayward, US.) with a paired-end 100 bp length configuration. Reads were mapped against the Human Reference Genome hg19 from UCSC Genome Browser (http://genome.ucsc.edu/) by using the Burrows-Wheeler Aligner (BWA) (version 0.7.6) [36]. Single nucleotide variations and indels were called using SAMtools (version 0.1.19) for single samples [37], and annotated using ANNOVAR [38].

### Analysis of whole exome sequencing data

The WES data were then analyzed following previously proposed guidelines [39,40]. The exomes of the four affected individuals were first screened for possible mutations, in any of the following twenty-three genes, previously reported to cause autosomal dominant cataracts: eleven crystallin genes, including *CRYAA* [MIM 123580] [41], *CRYAB* [MIM 123590] [42], *CRYBA1* [MIM 123610] [43], *CRYBA2* [MIM 600836] [5], *CRYBA4* [MIM 123631] [44], *CRYBB1* [MIM 600929] [45], *CRYBB2* [MIM 123620] [46], *CRYGB* [MIM 123670] [47], *CRYGC* [MIM 123680] [48], *CRYGD* [MIM 123690] [49], *CRYGS* [MIM 123730] [50]; three cytoskeletal protein genes: *BFSP1* [MIM 603307] [51], *BFSP2* [MIM 603212] [52] and VIM [MIM 193060] [53]; three membrane protein genes: *GJA3* [MIM 121015] [54], *GJA8* [MIM 600897] [55], *MIP* [MIM 154050] [56]; three growth and transcription factor genes, including *HSF4* [MIM 602438] [57], *PITX3* [MIM 602669] [58], *MAF* [MIM 177075] [59]; and three other genes, including *CHMP4B* [MIM 610897] [19], *EPHA2* [MIM 176946] [60], and *WFS1* [MIM 606201] [61].

Upon confirmation of no known autosomal dominant cataract gene mutations, the exomes were analyzed using step-by-step filtering strategies (**Fig 1D**). The estimated fraction of shared variants by descent was calculated using the identity-by-descent method implemented by MendelScan version 1.2.1 (http://gmt.genome.wustl.edu/mendelscan) [11] according to the autosomal dominant inheritance in Family 1. Genetic variants were first filtered to retain only non-synonymous single nucleotide variants, splicing site variants and coding indels. Second, filtering was performed to keep only variants with a minor allele frequency (MAF) < 0.1%, a reasonable cutoff for autosomal dominant diseases [62,63]. The variant MAFs were estimated using available data from dbSNP138 (http://www.ncbi.nlm.nih.gov/snp/), the 1000 Genomes Project (http://www.1000genomes.org), the NHLBI Exome Sequencing Project (ESP, http://evs.gs.washington.edu/EVS/), the exome array design data (http://genome.sph.umich.edu/wiki/Exome_Chip_Design) [64], and the Exome Aggregation Consortium (ExAC, http://exac.broadinstitute.org/), and KAVIAR data [65]. Third, remaining variants were filtered against a set of 125 in-house control exomes from 23 unrelated families and 67 unrelated individuals that were clinically examined and confirmed to have no sign or family history of congenital or juvenile-onset cataracts. Fourth, we kept only rare heterozygous variants that were shared between the four affected individuals of Family 1. Fifth, we used SIFT (http://genetics.bwh.harvard.edu/pph/) [66] and PolyPhen (http://sift.jcvi.org/www/SIFT_chr_coords_indels_submit.html) [67] programs to predict the functional impact of remaining variants on the encoded protein. Variants predicted as deleterious by either program was considered as candidate variants. Finally, Sanger sequencing was performed to confirm the candidate variants in all individuals with DNA samples available and to test for familial co-segregation of disease phenotype.

### Parametric linkage analysis

Linkage scores were calculated by MERLIN 1.1.2 [10]. Parametric analyses were performed to yield LOD sores, respectively. In the parametric analysis, the disease allele frequency was set to 0.0001% and penetrance with 0, 1 and 2 copies of the disease allele set to 0.01%, 100% and 100%, respectively.

### Sanger sequencing

The genomic sequence of genes with variants was obtained from the NCBI reference sequence database (http://www.ncbi.nlm.nih.gov/Refseq). Primers designed accordingly by Primer 3 are summarized in **S5 Table**. Polymerase chain reaction (PCR) amplification was performed using a GeneAmp PCR System 9700 (ABI, Foster City, CA) as described previously [9]. Sequence alignment and analysis of variations were performed using the NovoSNP program [68].

### In-house control exomes

A set of 125 in-house control exomes was used to filter out variants that were not related to the disease phenotype. The data set was consolidated from several previous WES studies performed by our laboratory. It contained 58 individuals from 23 unrelated families, and 67 unrelated individuals, all of whom had no sign or family history of congenital or juvenile-onset cataracts, and were recruited using the same criteria as described above after comprehensive ophthalmic examination at our eye center. Various exome capture chips were used for this data set, such as the Roche NimbleGen SeqCap EZ Human Exome Library v3.0 (Roche NimbleGen, Madison, US.), Agilent SureSelect All Human Exon v4.0 kit (Santa Clara, US.), or Ilumina TruSeq Exome Enrichment kit (Illumina, Hayward, US.). Sequencing was performed using an Illumina Hiseq 2000. The data quality criteria followed the same standard as we used for Family 1.

### Reverse transcription and PCR

Fresh primary human lens capsules were collected from senile cataract patients who received cataract surgery, and HLE were isolated. RNA was extracted from primary HLE and B-3 immortalized human lens epithelial cells with the NucleoSpin RNA kit (MACHEREY-NAGEL). A reverse transcription (RT) reaction was performed using 500 ng of total RNA with Takara PrimeScript RT Reagent Kit (Clonetech). The cDNA sequences of PCR products were confirmed using Sanger sequencing. Primers for expression analysis are listed in **S5 Table**.

### Homology remodeling of protein structure

The three-dimensional structure of the RRAGA protein was obtained by means of homology modeling using Swiss-PdbViewer version 4.1. The crystallographic structure at 2.77-Å resolution of GTP-binding protein (GTR1) from Saccharomyces cerevisiae, in complex with Mg^2+^ and GTP (PDB entry 3r7w chain A), was used as the template[12]. The selected template ensured a sequence identity of 55.05% and a global model quality estimation (GMQE) score of 0.73. The coordinates of the Mg^2+^ and GTP backbone were taken from the crystal structure of the two molecules bound to GTR1.

### Antibodies

Antibodies used in this study include the following: rabbit monoclonal anti-RRAGA (Cell Signaling Technology, 4357); rabbit monoclonal anti-CRYAB (GeneTex, GTX61997); rabbit monoclonal anti-mTOR (Cell Signaling Technology, 2983); rabbit monoclonal anti-p-mTOR (Ser2448) (Cell Signaling Technology, 5536); HRP-conjugated mouse monoclonal anti-GAPDH (Kangchen, KC-5G5); mouse monoclonal anti-GFP (Santa Cruz, SC-9996); mouse monoclonal anti-luciferase (LSBio, LS-C71819); Alexa Fluor 546 donkey anti-rabbit IgG (Life Technologies, A10040); and Alexa Fluor 488 goat anti-mouse IgG (Life Technologies, A-11001); HRP-conjugated goat anti-rabbit IgG (BioRad, 170–6515).

### Plasmids

The human *RRAGA* gene contains only a single exon without any intron, and thus its cDNA and genomic DNA have exactly the same sequence. The full length coding sequences of *RRAGA* wild-type and mutant with c.157A>G (p.Leu60Arg) were amplified from the genomic DNA of unaffected control III-7 and affected patient III-2 from Family 1 respectively, by using PCR, and inserted into the pEGFP-C1 expression vector (Clontech, Mountain view, U.S.) between the HindIII and BglII restriction sites respectively. The *RRAGA* 5’-UTR region (1 to 286 bp upstream from the start codon) of wild-type 5’-UTR and the c.-16G>A mutant was amplified from unaffected control III-7 and affected patient CC19 respectively, and inserted into the pGL3-enhancer vector (Promega, Madison, U.S.) between the HindIII and BglII restriction sites. Sequences of constructs were confirmed using Sanger sequencing. Primers used for plasmid construction are listed in **S4 Table**.

### Expression of RRAGA protein in the human lens

Normal human eyeballs (*n* = 3), from deceased individuals, were obtained from the archive of Ocular Pathology Service, the Chinese University of Hong Kong. The localization of RRAGA protein was determined by immunofluorescence analysis. Whole eyeballs were fixed in 10% formalin and embedded in paraffin. Paraffin sections (5 μm thick) were first de-waxed and re-hydrated in sequential incubation in xylene and ethanol. Antigen was retrieved in Tris-EDTA buffer at 80°C for 20 minutes. After permeation and blocking in 0.425% saponin, 0.0015% Triton-X 100, 0.0015% Tween-20, 1% bovine serum albumin and 1% normal goat serum, eyeball sections were labeled with primary rabbit polyclonal antibodies against RRAGA at 4°C for 16–18 hr and secondary antibodies against rabbit IgG, conjugated with Alexa Fluor 488 (Santa Cruz Biotechnology), at room temperature for 1 hr. Fluorescence was visualized under a fluorescence microscope (Eclipse Ni-U, Nikon).

### Cell culture and transfection

Human lens epithelial B-3 cells (American Tissue Culture Collection, Manassas, VA) were cultured in 6-well plates (Corning) in Dulbecco’s modified Eagle’s medium: Ham’s F-12 (DMEM/F12, Gibco) supplemented with 10% fetal bovine serum (FBS, Gibco) and antibiotics (100 units/ml penicillin G and 100 μg/ml streptomycin sulfate, Gibco) at 37°C in a 5% CO_2_ incubator. For GFP overexpression, cells were transfected with 6 μg of plasmid and Lipofectamine 2000 (Invitrogen). For LAMP1 analysis, B-3 cells were transfected with CellLight Lysosomes-RFP, BacMam 2.0 (Life Technologies) following the manufacturer's instructions.

### Immunofluorescence staining and confocal microscopy

The cells were inoculated on glass bottom culture dishes (MatTek Co, Ashland, U.S.) in DMEM/F12 supplemented with 10% FBS, and grown for 20 hr. Cells were then fixed with 3% paraformaldehyde for 5 min, followed by 0.5% paraformaldehyde for 30 min, and then washed with PBS. Fixed cells were permeabilized with Triton X-100 and saponin for 10 min at room temperature. For immunofluorescence staining, cells were incubated with the appropriate primary antibodies followed by fluorescence-conjugated secondary antibodies and 4'-6-diamidino-2-phenylindole (DAPI; Sigma, St. Louis, U.S.). Confocal microscopy was performed on a Leica TCS SP5 confocal microscope.

### Cell area analysis

Images of transfected cells captured under a 10x objective lens were analyzed using ImageJ version 2.0. The green fluorescence signal was converted into binary images according to a threshold determined by the mean algorithm implemented by the software. Cells were then detected and cell area was calculated automatically by using the “Analyze particles” function. Only particles with areas of 80–1000 μM^2^ were analyzed to ensure accuracy in automated cell detection.

### Immunoblotting

B-3 cells were lysed and total protein was extracted using a NucleoSpin Protein kit (MACHEREY-NAGEL, Düren, Germany) supplemented with a protease inhibitor tablet (Roche, Basel, Switzerland). Protein samples were run on a 12% acrylamide gel, and immunoblotting was performed using antibodies described above.

### Immunoprecipitation

Transfected cells were lysed, and total protein (input) was subjected to immunoprecipitation with GFP-Trap_A (Chromtek, Planegg-Martinsried, Germany), followed by immunoblotting analysis with anti-GFP antibodies.

### Statistical analysis

The Mann-Whitney U test was used to compare cell area between different groups of transfected cells. The Student’s t-test was used to compare quantification data of immunoblotting between different groups of samples.

## Supporting Information

S1 Fig Sanger sequencing confirmation of co-segregation between *RRAGA* p.Leu60Arg mutation and juvenile onset cataracts in Family 1. The left panel shows chromatography for the heterozygous *RRAGA* c.179T>G (p.Leu60Arg) that was detected in nine affected individuals in Family 1 with juvenile onset progressive posterior subcapsular cataracts. The upper-right panel shows the homozygous wild-type genotype in three unaffected family members. The lower right panel shows the homozygous wild-type genotype observed in all 1018 unaffected unrelated controls.
(PDF)

S2 Fig Sanger sequencing of *RRAGA* mutations in unrelated patients. The left panel shows chromatography for the heterozygous mutations found in unrelated patients with congenital (CC19) or juvenile onset cataracts (CC38). The right panel shows the homozygous wild-type genotypes in 1018 unaffected controls.
(PDF)

S3 Fig Lens photos of patients with *RRAGA* p.Leu60Arg mutation. (PDF)

S4 Fig Expression of RRAGA in B-3 human lens epithelial cells. (**A**) Quantification of *RRAGA*, *FYCO1* and *CRYAB* expression using qPCR. Expression level of *RRAGA* is normalized with GAPDH. (**B**) Western blotting of RRAGA protein in B-3 cells.
(PDF)

S5 Fig Immunofluorescence of RRAGA in human retina and cornea. RRAGA (green) can be observed with lower expression in the retina (**A**) and with the lowest expression in the cornea (**B**) compared to the lens epithelia. DNA is stain with DAPI (blue).
(PDF)

S1 Table Summary of original exome sequencing data of Family 1 with juvenile-onset progressive posterior subcapsular cataracts. (PDF)

S2 Table Summary of detected variants in four exomes of Family 1 with juvenile-onset progressive posterior subcapsular cataracts. (PDF)

S3 Table Summary of sequencing depths in coding exons of the twenty-three autosomal dominant cataract genes in the four exomes of Family 1. (PDF)

S4 Table Sequences of primers. (PDF)

S5 Table Results of Sanger sequencing validation of candidate variants in Family 1. (PDF)

S6 Table Haplotypes in in the four exomes of Family 1 and the unrelated simplex SCC38. ^a^10 Coding SNVs that are heterozygous in at least one of the affected are selected to construct a haplotype (in bold face) that contains the alternative allele G of *RRAGA* c.179T>G. ^d^Differences are found between haplotypes that contain contains the alternative allele G of *RRAGA* c.179T>G in Family 1 and SCC38.
(PDF)
