# Supplementary material for: Mutations of RagA GTPase in mTORC1 Pathway Are Associated with Autosomal Dominant Cataracts
Source: PLoS Genet. 2016 Jun 13;12(6):e1006090. doi: 10.1371/journal.pgen.1006090 (PMC4905677; doi:10.1371/journal.pgen.1006090)
Supplement: S1 Fig — The left panel shows chromatography for the heterozygous RRAGA c.179T>G (p.Leu60Arg) that was detected in nine affected individuals in Family 1 with juvenile onset progressive posterior subcapsular cataracts. The upper-right panel shows the homozygous wild-type genotype in three unaffected family members. The lower right panel shows the homozygous wild-type genotype observed in all 1018 unaffected unrelated controls. (PDF) [file pgen.1006090.s001.pdf]

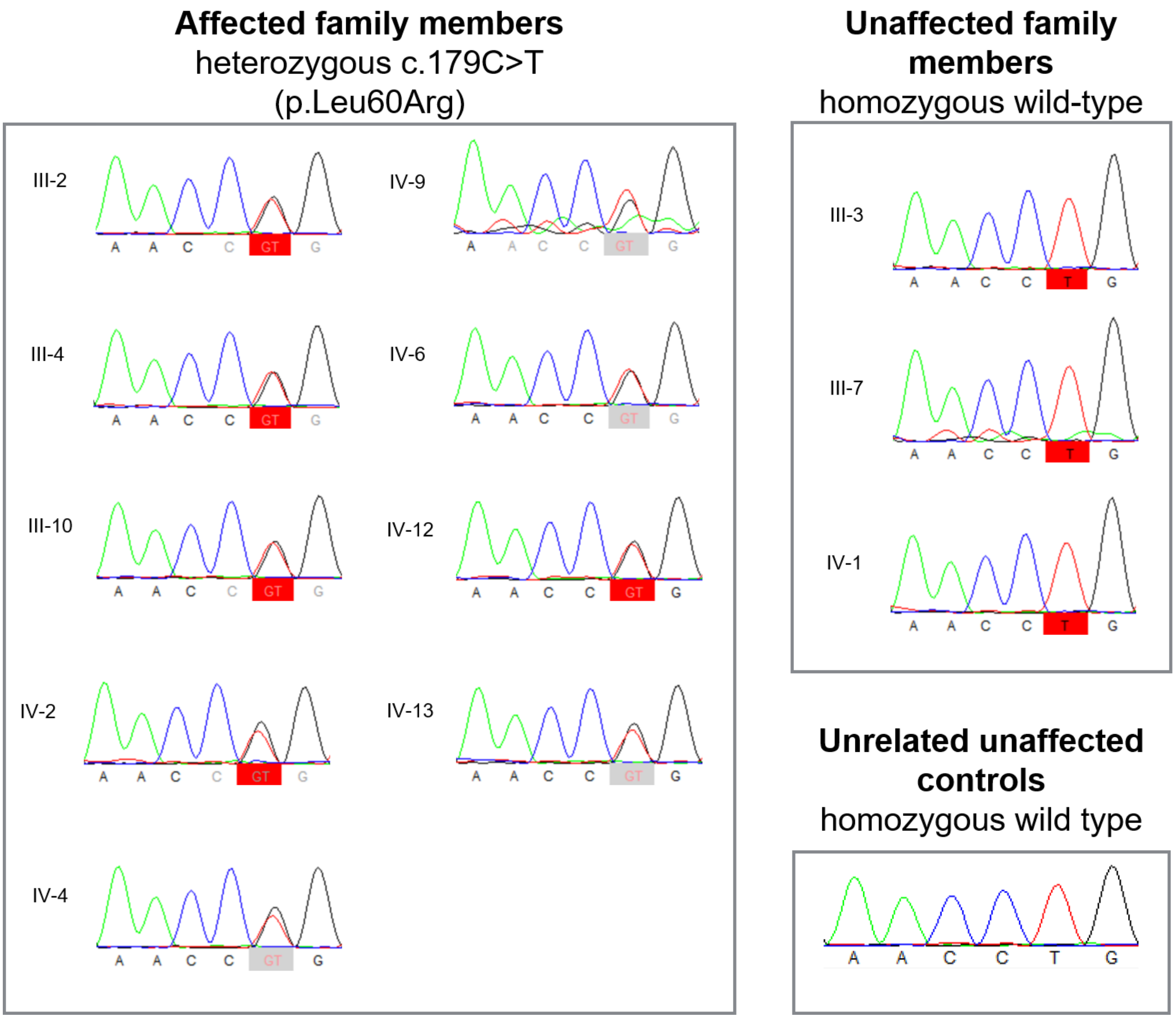

S1 Fig. Sanger sequencing confirmation of co-segregation between *RRAGA* p.Leu60Arg mutation and juvenile onset cataracts in Family 1. The left panel shows chromatography for the heterozygous *RRAGA* c.179T>G (p.Leu60Arg) that was detected in nine affected individuals in Family 1 with juvenile onset progressive posterior subcapsular cataracts. The upper-right panel shows the homozygous wild-type genotype in three unaffected family members. The lower right panel shows the homozygous wild-type genotype observed in all 1018 unaffected unrelated controls.
