# Supplementary material for: Mutations of RagA GTPase in mTORC1 Pathway Are Associated with Autosomal Dominant Cataracts
Source: PLoS Genet. 2016 Jun 13;12(6):e1006090. doi: 10.1371/journal.pgen.1006090 (PMC4905677; doi:10.1371/journal.pgen.1006090)
Supplement: S2 Fig — The left panel shows chromatography for the heterozygous mutations found in unrelated patients with congenital (CC19) or juvenile onset cataracts (CC38). The right panel shows the homozygous wild-type genotypes in 1018 unaffected controls. (PDF) [file pgen.1006090.s002.pdf]

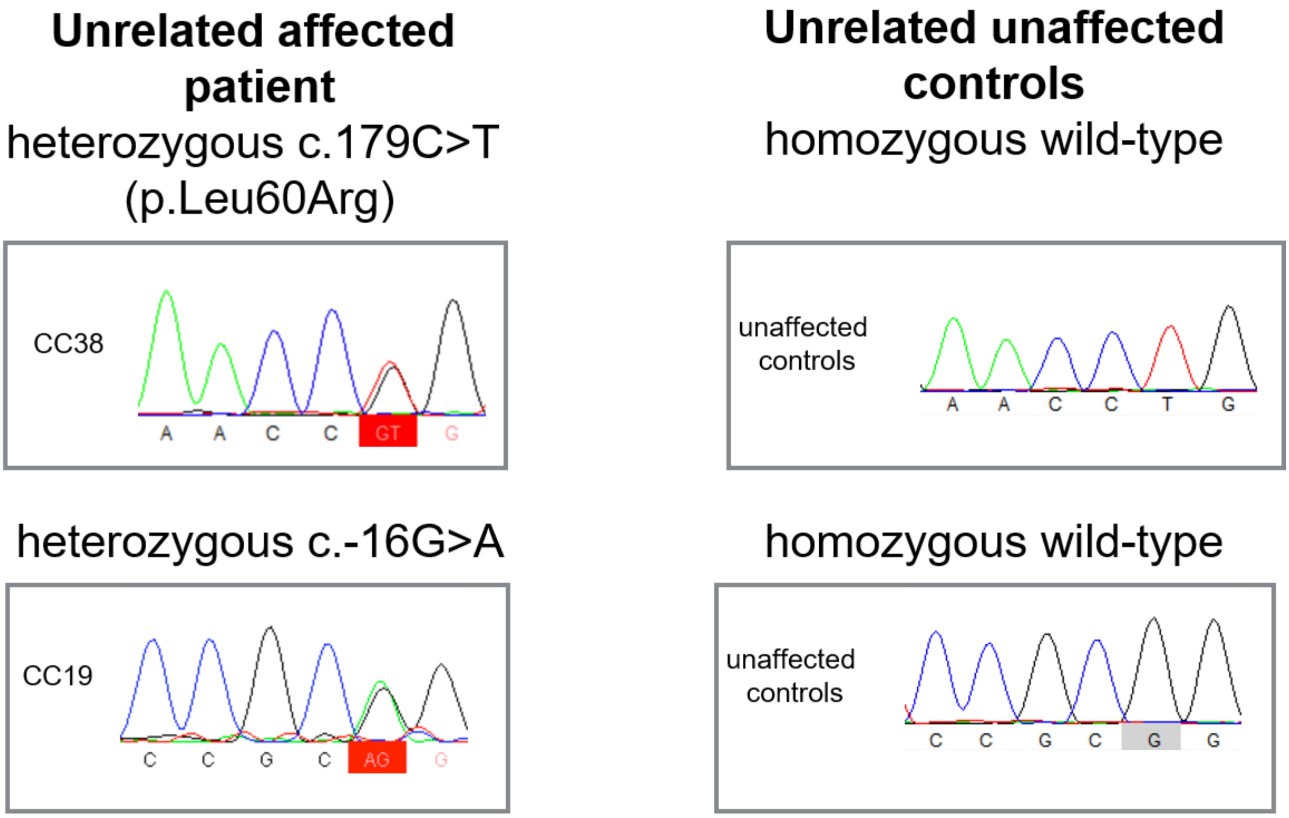

S2 Fig. Sanger sequencing of *RRAGA* mutations in unrelated patients. The left panel shows chromatography for the heterozygous mutations found in unrelated patients with congenital (CC19) or juvenile onset cataracts (CC38). The right panel shows the homozygous wild-type genotypes in 1018 unaffected controls.
