# Supplementary figures and images for: Mutations of RagA GTPase in mTORC1 Pathway Are Associated with Autosomal Dominant Cataracts
Source: PLoS Genet. 2016 Jun 13;12(6):e1006090. doi: 10.1371/journal.pgen.1006090 (PMC4905677; doi:10.1371/journal.pgen.1006090)

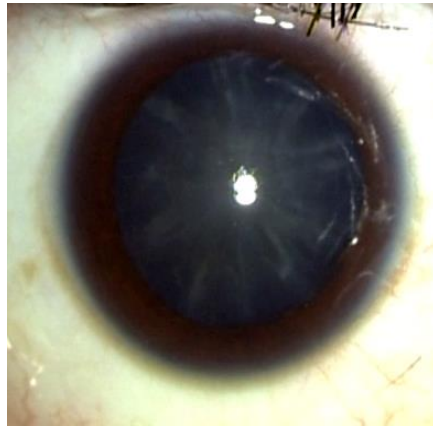

**IV-12**

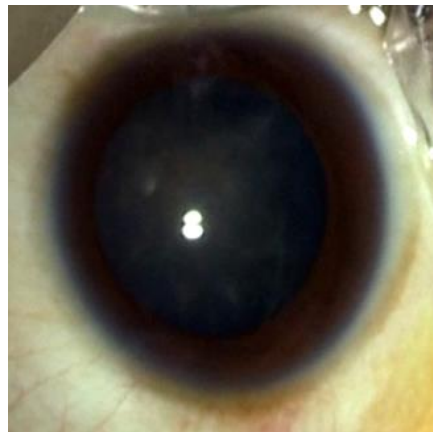

**IV-9**

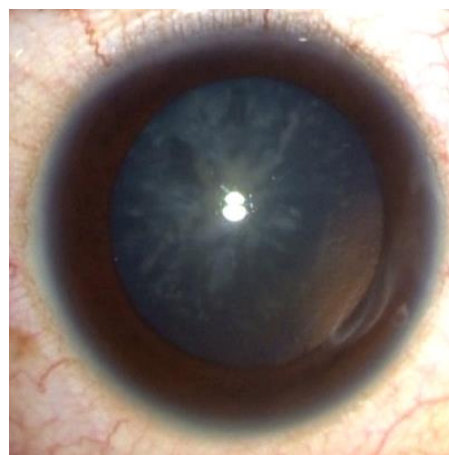

**CC38**

**S3 Fig. Lens photos of patients with *RRAGA* p.Leu60Arg mutation.**

Supplement: S3 Fig — (PDF) [file pgen.1006090.s003.pdf]
