# Supplementary material for: Mutations of RagA GTPase in mTORC1 Pathway Are Associated with Autosomal Dominant Cataracts
Source: PLoS Genet. 2016 Jun 13;12(6):e1006090. doi: 10.1371/journal.pgen.1006090 (PMC4905677; doi:10.1371/journal.pgen.1006090)
Supplement: S4 Fig — (A) Quantification of RRAGA, FYCO1 and CRYAB expression using qPCR. Expression level of RRAGA is normalized with GAPDH. (B) Western blotting of RRAGA protein in B-3 cells. (PDF) [file pgen.1006090.s004.pdf]

A

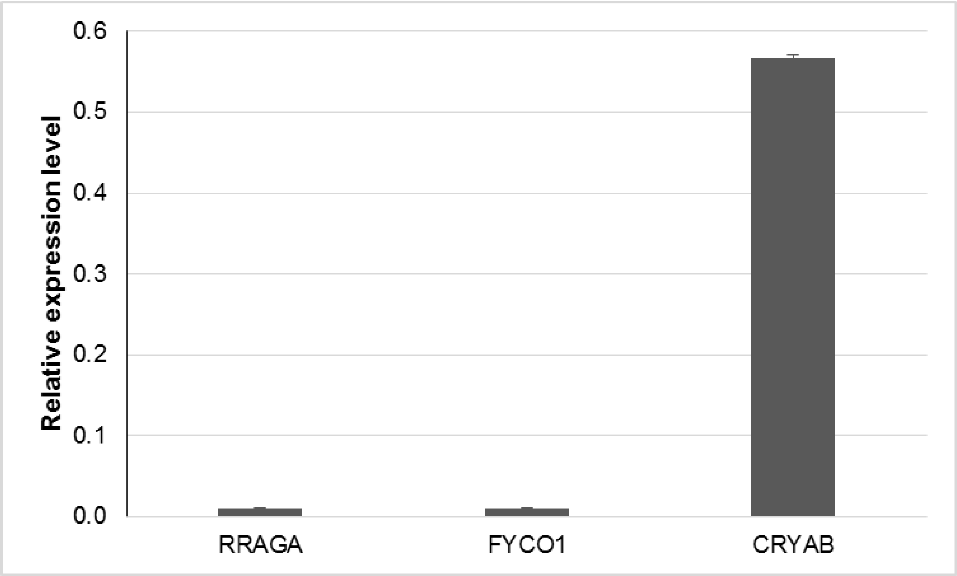

B

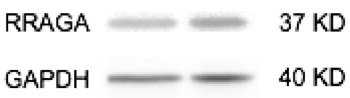

S4 Fig. Expression of RRAGA in B-3 human lens epithelial cells. (**A**) Quantification of *RRAGA*, *FYCO1* and *CRYAB* expression using qPCR. Expression level of *RRAGA* is normalized with GAPDH. (**B**) Western blotting of RRAGA protein in B-3 cells.
