# Supplementary material for: Mutations of RagA GTPase in mTORC1 Pathway Are Associated with Autosomal Dominant Cataracts
Source: PLoS Genet. 2016 Jun 13;12(6):e1006090. doi: 10.1371/journal.pgen.1006090 (PMC4905677; doi:10.1371/journal.pgen.1006090)
Supplement: S5 Fig — RRAGA (green) can be observed with lower expression in the retina (A) and with the lowest expression in the cornea (B) compared to the lens epithelia. DNA is stain with DAPI (blue). (PDF) [file pgen.1006090.s005.pdf]

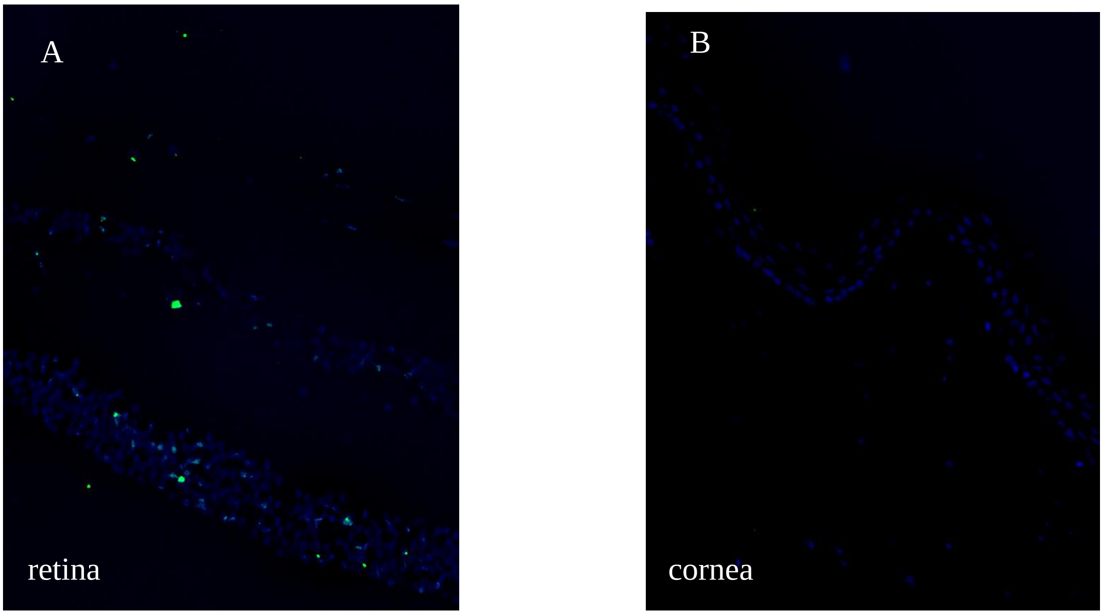

S5 Fig. Immunofluorescence of RRAGA in human retina and cornea. RRAGA (green) can be observed with lower expression in the retina (**A**) and with the lowest expression in the cornea (**B**) compared to the lens epithelia. DNA is stain with DAPI (blue).
