# Supplementary material for: Mutations of RagA GTPase in mTORC1 Pathway Are Associated with Autosomal Dominant Cataracts
Source: PLoS Genet. 2016 Jun 13;12(6):e1006090. doi: 10.1371/journal.pgen.1006090 (PMC4905677; doi:10.1371/journal.pgen.1006090)
Supplement: S1 Table — (PDF) [file pgen.1006090.s006.pdf]

S1 Table. Summary of original exome sequencing data of Family 1 with juvenile onset progressive posterior subcapsular cataracts.

Data	III-2	IV-9	IV-12	IV-13	Mean
Number of raw reads (M)	56.9	47.8	53.1	48.9	51.6
Average read length (bp)	101	101	101	101	101
Raw data yield (Gb)	5.8	4.8	5.4	4.9	5.2
Number of reads mapped to the genome (M)	56.6	47.7	52.9	48.6	51.3
Fraction of uniquely mapped bases on target (%)	80.6%	80.0%	80.6%	77.0%	79.3%
Data mapped to target region (Gb)	4.2	3.6	4.1	3.6	3.9
Mean depth of target region (fold)	72.3	60.2	68.3	60.8	65.1
Coverage of target region (%)	99.0%	99.1%	99.0%	98.9%	99.0%
Target region with more than 10X (%)	96.3%	97.0%	95.6%	94.6%	95.8%
