# Supplementary material for: Mutations of RagA GTPase in mTORC1 Pathway Are Associated with Autosomal Dominant Cataracts
Source: PLoS Genet. 2016 Jun 13;12(6):e1006090. doi: 10.1371/journal.pgen.1006090 (PMC4905677; doi:10.1371/journal.pgen.1006090)
Supplement: S2 Table — (PDF) [file pgen.1006090.s007.pdf]

S2 Table. Summary of detected variants in the four exomes of Family 1 with juvenile onset progressive posterior subcapsular cataracts.

Variants	III-2	IV-9	IV-12	IV-13	Mean
Number of SNPs	64136	65499	64796	63769	64550
Number of coding SNPs	19406	19536	19719	19631	19620
Number of synonymous SNPs	10166	10318	10391	10391	10325
Number of nonsynonymous SNPs	8802	8760	8840	10249	9123
Number of Indels	5684	6167	5685	5494	5718
Number of coding Indels	468	464	486	478	472
