# Supplementary material for: Mutations of RagA GTPase in mTORC1 Pathway Are Associated with Autosomal Dominant Cataracts
Source: PLoS Genet. 2016 Jun 13;12(6):e1006090. doi: 10.1371/journal.pgen.1006090 (PMC4905677; doi:10.1371/journal.pgen.1006090)
Supplement: S3 Table — (PDF) [file pgen.1006090.s008.pdf]

S3 Table. Summary of sequencing depths in coding exons of the twenty-three autosomal dominant cataract genes in the four exomes of Family 1.

Individual	Min	Median	Mean	Max
**III-2**	10	77	81	195
**IV-9**	5	58	61	142
**IV-12**	9	73	80	195
**IV-13**	10	63	70	170
