# Supplementary material for: Mutations of RagA GTPase in mTORC1 Pathway Are Associated with Autosomal Dominant Cataracts
Source: PLoS Genet. 2016 Jun 13;12(6):e1006090. doi: 10.1371/journal.pgen.1006090 (PMC4905677; doi:10.1371/journal.pgen.1006090)
Supplement: S4 Table — (PDF) [file pgen.1006090.s009.pdf]

S4 Table. Sequences of primers.

No.	Primer	Gene symbol	Exon	Primer Sequence	Used in
1	RRAGA-L60F<br>RRAGA-L60R	RRAGA	1	TGCTGCTGATGGGGAAGAG<br>ATGTCCTTTTCCAGTTCGCG	Family 1
2	RRAGA-1F<br>RRAGA-1R	RRAGA	1	TCTGAGTAAGAGCCAGCCC<br>CAGGCAGAAGATTTTGGCGT	Families and unrelated patients; controls
3	RRAGA-2F<br>RRAGA-2R	RRAGA	1	ACCAGCCAGCGAGACAATAT<br>TCGAACAGCAGAACTTCATCG	Families and unrelated patients; controls
4	RRAGA-3F<br>RRAGA-3R	RRAGA	1	TGATTCCCAACGTTCAGCAG<br>GCCCACTTTTAAGCACACGA	Families and unrelated patients; controls
5	KIF27F<br>KIF27R	KIF27	4	GGCTGGTGTCAGCCAAACTA<br>GTGCCTCCGATTCCTCGAAAT	Families and unrelated patients; controls
6	ZNF48F<br>ZNF48R	ZNF48	3	CCTGAAGGCATCCAGAACTG<br>AAACCTCTTGCCGCAATCAG	Families and unrelated patients; controls
7	PEG3F<br>PEG3R	PEG3	5	TGTGGGAAGAGGGTAGCATC<br>GAGCAGCAGAAACTTCGAGG	Family 1
RT-PCR					
9	RRAGAF<br>RRAGAR	RRAGA		ACCAGCCAGCGAGACAATAT<br>TCGAACAGCAGAACTTCATCG	
10	CRYABF<br>CRYABR	CRYAB		GGCAAAGAGCAGCTCAGTGAGTA<br>ATCCTGGCGCTCTTCATGTT	
11	FYCO1F<br>FYCO1R	FYCO1		TCCTCTTTGCCTGAAACACC<br>GGGCATGTCTTCAGTGTCCT	
12	GAPDHF<br>GAPDHR	GAPDH		GAAGGTGAAGGTCGGAGTCA<br>CCTGGAAGATGGTGATGGGA	
RRAGA expression					
12	RRAGA- CDSF<br>RRAGA- CDSR	RRAGA		CTCGGCATGGACGAGCTGTACAAGTCCGGACTCAGATCTATGCCAAATACAGCC<br>CCGGGCCCGCGGTACCGTCGACTGCAGAATTCGAAGCTTTCAACGCATAAGGAG	III-2 in family 1
RRAGA 5'-UTR promoter activity					
13	RRAGA-5UTRF<br>RRAGA-5UTRR	RRAGA		GTACCGAGCTCTTACGCGTGCTAGCCCGGGCTCGAGATCTCGCCGGAAGTGGGTG<br>CCATGGTGGCTTTACCAACAGTACCGGAATGCCAAGCTTCACCCGCCGGGGCAC	CC19
