# Supplementary material for: Mutations of RagA GTPase in mTORC1 Pathway Are Associated with Autosomal Dominant Cataracts
Source: PLoS Genet. 2016 Jun 13;12(6):e1006090. doi: 10.1371/journal.pgen.1006090 (PMC4905677; doi:10.1371/journal.pgen.1006090)
Supplement: S5 Table — (PDF) [file pgen.1006090.s010.pdf]

S5 Table. Results of Sanger sequencing validation of candidate variants in Family 1.

Individual	Status	Genotype			
		KIF27	***ZNF48***	***PEG3***	***RRAGA***
		**c.1253C>G**	**c.503A>G**	**c.499C>T**	**c.179T>G**
III:2	affected	C/G	A/G	C/T	G/T
III:3	unaffected	C/C	A/A	C/C	T/T
III:4	affected	C/G	A/G	C/C	G/T
III:7	unaffected	C/C	A/A	C/C	T/T
III:10	affected	C/G	A/G	C/T	G/T
IV:1	unaffected	C/G	A/G	C/C	T/T
IV:2	affected	C/G	A/A	C/T	G/T
IV:4	affected	C/C	A/G	C/C	G/T
IV:9	affected	C/G	A/G	C/T	G/T
IV:12	affected	C/G	A/G	C/T	G/T
IV:13	affected	C/G	A/G	C/T	G/T
