# Supplementary material for: Mutations of RagA GTPase in mTORC1 Pathway Are Associated with Autosomal Dominant Cataracts
Source: PLoS Genet. 2016 Jun 13;12(6):e1006090. doi: 10.1371/journal.pgen.1006090 (PMC4905677; doi:10.1371/journal.pgen.1006090)
Supplement: S6 Table — a10 Coding SNVs that are heterozygous in at least one of the affected are selected to construct a haplotype (in bold face) that contains the alternative allele G of RRAGA c.179T>G. dDifferences are found between haplotypes that contain contains the alternative allele G of RRAGA c.179T>G in Family 1 and SCC38. (PDF) [file pgen.1006090.s011.pdf]

S6 Table. Haplotypes in in the four exomes of Family 1 and the unrelated simplex SCC38.

			III-2		IV-9		IV-12		IV-13		SCC38	
**Chromosome**	**Position**	**Variant[a]**	**haplotype1**	**haplotype2**	**haplotype1**	**haplotype2**	**haplotype1**	**haplotype2**	**haplotype1**	**haplotype2**	**haplotype1**	**haplotype2**
9	18777422	rs1549986	G	**G**	G	**G**	A	**G**	A	**G**	A	A[b]
9	18928312	rs7033684	C	**C**	G	**C**	C	**C**	C	**C**	C	C
9	18950787	rs6475273	A	**A**	G	**A**	A	**A**	A	**A**	A	A
9	18950895	rs7021572	C	**C**	T	**C**	C	**C**	C	**C**	C	C
9	19049836	RRAGA c.179T>G	T	**G**	T	**G**	T	**G**	T	**G**	T	G
9	19058743	rs10511670	C	**G**	C	**G**	G	**G**	G	**G**	G	G
9	19063108	rs13302748	C	**T**	C	**T**	T	**T**	T	**T**	C	C[b]
9	19076673	rs13286328	C	**C**	C	**C**	C	**C**	C	**C**	C	C
9	19076692	rs559195154	A	**A**	A	**A**	A	**A**	A	**A**	A	A
9	19516280	rs3739480	G	**G**	G	**G**	G	**G**	G	**G**	G	G

[a]10 Coding SNVs that are heterozygous in at least one of the affected are selected to construct a haplotype (in bold face) that contains the alternative allele G of *RRAGA* c.179T>G.

[b]Differences are found between haplotypes that contain contains the alternative allele G of *RRAGA* c.179T>G in Family 1 and SCC38.
